# Abnormal dewetting of Ag layer on three-dimensional ITO branches to form spatial plasmonic nanoparticles for organic solar cells

**DOI:** 10.1038/s41598-020-69320-4

**Published:** 2020-07-30

**Authors:** Wan Jae Dong, Hak Ki Yu, Jong-Lam Lee

**Affiliations:** 1 Department of Materials Science and Engineering, Pohang University of Science and Technology (POSTECH), Pohang, 790-784 Republic of Korea; 2 Department of Materials Science and Engineering and Department of Energy Systems Research, Ajou University, Suwon, 16499 Republic of Korea

**Keywords:** Nanoscale materials, Solar cells, Nanophotonics and plasmonics

## Abstract

Three-dimensional (3D) plasmonic structures have attracted great attention because abnormal wetting behavior of plasmonic nanoparticles (NPs) on 3D nanostructure can enhance the localized surface plasmons (LSPs). However, previous 3D plasmonic nanostructures inherently had weak plasmonic light absorption, low electrical conductivity, and optical transmittance. Here, we fabricated a novel 3D plasmonic nanostructure composed of Ag NPs as the metal for strong LSPs and 3D nano-branched indium tin oxide (ITO BRs) as a transparent and conductive framework. The Ag NPs formed on the ITO BRs have a more dewetted behavior than those formed on the ITO films. We experimentally investigated the reasons for the dewetting behavior of Ag NPs concerning the geometry of ITO BRs. The spherical Ag NPs are spatially separated and have high density, thereby resulting in strong LSPs. Finite-domain time-difference simulation evidenced that spatially-separated, high-density and spherical Ag NPs formed on ITO BRs dramatically boost the localized electric field in the active layer of organic solar cells (OSCs). Photocurrent of PTB7:PCBM OSCs with the ITO BRs/Ag NPs increased by 14%.

## Introduction

Controlling the wettability of metal on target surfaces such as oxides and polymer is one of the most classical challenges in many theoretical and engineering fields because the morphology of metal film is closely related to the device performance and stability^1–4^. The basic solution of the wetting phenomena can be explained by Young’s theory based on the thermo-dynamical equilibrium between metal, surface, and atmosphere^5,6^. However, the theory has difficulty to explain the abnormal wetting behavior at nanoscale ranges where a large surface to volume ratio and strong cohesive energy should be considered. Recently, several calculations and experimental results have been reported that the additional line tension of nanoscale particles on the surface can increase the contact angle, resulting in a more spherical type of wetting^7–10^.

The wetting behavior of metal nanoparticles (NPs) with high electron density such as Ag, Au, and Al have been extensively studied for the applications of localized surface plasmons (LSPs), such as light absorption in organic solar cells (OSCs)^11–45^. To maximize the effects of LSPs in the active layer of OSCs, the shape of NPs are important^11–13^. So, various geometric structures of metal nanowires^23^, nanoclusters^24^, nanocubes^17,18,37^, and nanoprisms^43^, have been studied using nanotechnology such as electron beam lithography^46^, UV-nanoimprint lithography^47^, etched anodic aluminum oxide^48^, and so on. However, many of these methods have limitations such as high-temperature process, high cost, reproducibility, and especially, complexity of fabrication. Moreover, the metal NPs can be agglomerated by forming cluster during device operation, resulting in degradation of device performances such as charge-carrier recombination, and exciton quenching^49,50^.

To overcome these problems, 3-dimensional (3D) plasmonic structures were demonstrated by attaching the plasmonic NPs on zinc oxide nanorods (ZnO NRs)^38,51^, titanium oxide (TiO_2_) NRs^22,28,52,53^, carbon nanotubes (CNTs)^26,54^ and polymeric nanofiber^20^. Because metal NPs were stuck on the nanostructures, phase separation or aggregation of NPs could be prevented. However, there are several issues needed to be addressed before the 3D plasmonic structures are applied to the device. (1) First, the previous 3D plasmonic structures had a wide gap between the NPs and had the morphology of a network of islands in which particles were not spatially seperated. Thus, the localized electric field was weak. (2) Second, the framework of ZnO, TiO_2_ NRs and CNTs inherently have low electrical conductivity and optical transmittance. To obtain high photocurrent in the OSCs, it is required to develop a new 3D plasmonic structure having strong plasmonic interaction as well as high electrical conductivity and optical transparency.

In this work, we used the randomly distributed three-dimensional (3D) nano-branches as a framework for thin metal film deposition to realize the abnormal wetting behavior of nano-sized metal. The thin metal film on 3D nano-branch substrate can be automatically disconnected without additional process, forming the metal NPs where the additional line tension should be considered. Because the nano-sized metal film could be transformed into liquid phase by mild-heating (the melting point of nano-size metal film is lower than the bulk state), wetting angle was increased, leading to spherical metal NPs (contact angle > 150°). We used the Ag as the metal film for strong LSPs and 3D nano-branched indium tin oxide (ITO BRs) as the framework because the surface migration of Ag can be easily controlled by low-temperature heating and the ITO is an ideal material for optoelectronic devices due to high electrical conductivity and optical transparency. The Ag NPs formed on the ITO BRs have a more dewetted behavior at low temperatures than that formed on the ITO film. We experimentally investigated the reasons for enhanced dewetting of Ag NPs concerning the geometry of ITO BRs. Because the spatially separated Ag NPs on 3D ITO BRs can boost the localized electric field, photocurrent in PTB7:PCBM organic solar cells (OSCs) enhanced by 14% when the 3D plasmonic structure was embedded in the active layer.

## Results and discussion

### Nucleation of Ag layer deposited on ITO BRs

**Figure 1:**
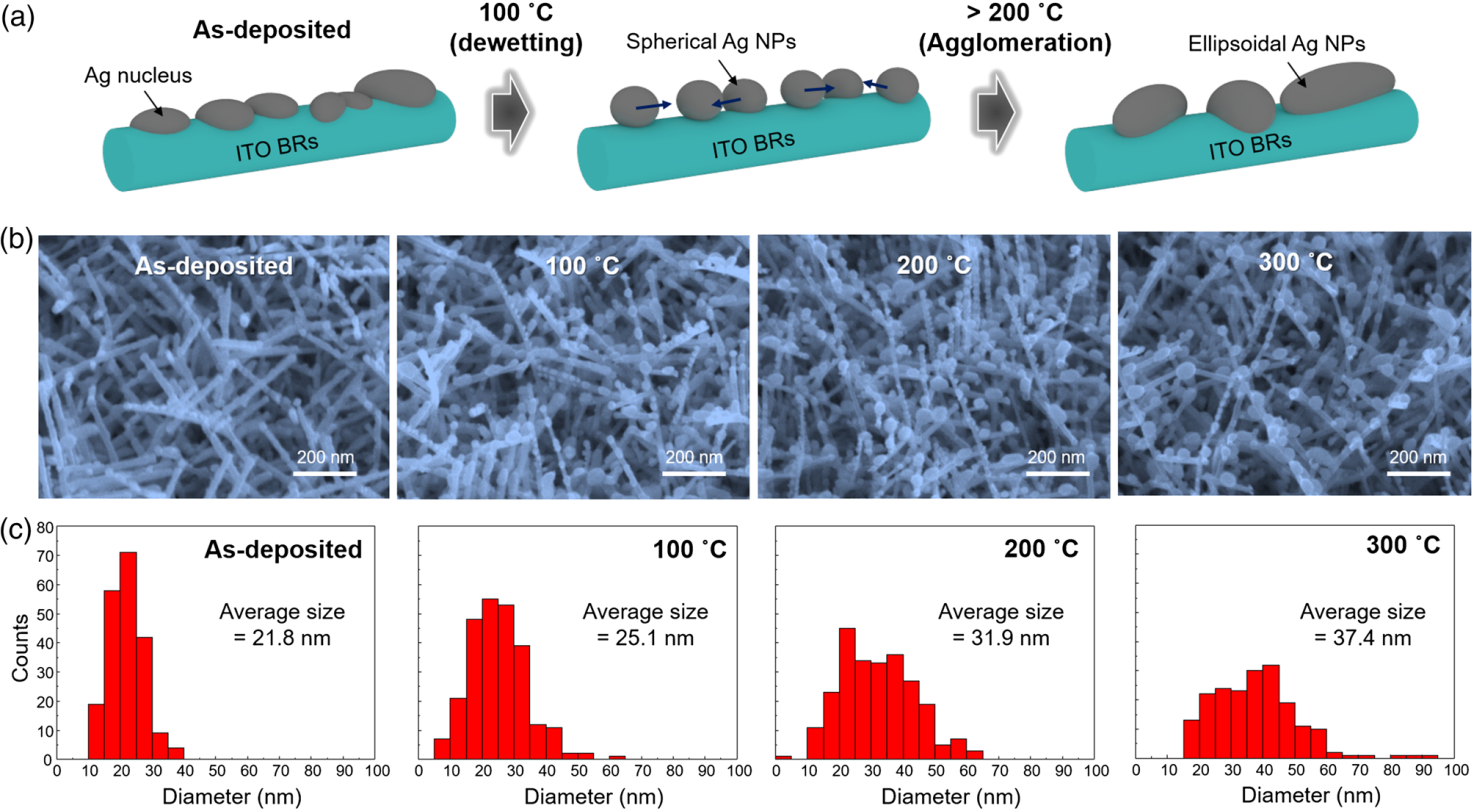
Dewetting of Ag NPs on ITO BRs. (**a**) Schematic illustrations of dewetting and agglomeration of Ag NPs on ITO BRs during thermal annealing. As-deposited Ag nuclei showed a particle-like shape on ITO BRs. Then, they were changed to spherical shape after annealing at 100 °C. Further increase in annealing temperature (> 200 °C) resulted in ellipsoidal Ag NPs due to agglomeration. (**b**) SEM images of ITO BRs/Ag NPs for as-deposited and thermally annealed samples. (**c**) Size distributions of Ag NPs on ITO BRs. As-deposited Ag nuclei had average diameter (D_avg_) = 21.8 nm. As the annealing temperature increased from 100 to 300 °C, D_avg_ gradually increased from 25.1 to 37.4 nm.

Ag NPs were prepared by depositing a thin Ag layer (10 nm) on the ITO BRs or ITO film, followed by thermal annealing (Fig. 1a). When the 10 nm-thick Ag layer was deposited on ITO BRs, the Ag layer showed discontinuous particles due to nucleation of Ag atoms (Fig. 1b). In contrast, the Ag layer on the ITO film showed a network of islands (Supplementary information Figure S1). One possible reason for the enhanced dewetting of the Ag layer is the 3D geometry of ITO BRs. Typically, thin metal layers deposited on vertically aligned nanostructures form particle-like nuclei on the sidewalls of nanostructures due to the shadowing effect^55^. So, the shadowing effect can enhance the formation of Ag NPs on ITO BRs. In addition, line-tension plays a critical role in the dewetting of nanoscale metal particles. In line-tension theory, the contact angle of a small droplet is affected by the size of the NPs as follows^9,10^:

$$\cos (\theta ) = \frac{{\gamma_{SV} - \gamma_{SL} }}{{\gamma_{LV} }} - \frac{\tau }{{\gamma_{LV} r}}$$

where *θ* is the contact angle of nanoparticle, γ_SV_, γ_LV_ are the surface energies of the substrate and droplet, γ_SL_ is the interfacial energy, *r* is the radius of the NP and τ is the interfacial line tension. Because the diameter of ITO BRs is small as about 30 nm, the size (r) of Ag NPs on ITO BRs is also smaller than that on the ITO film. So, the line-tension (τ/γ_LV·_*r*) of Ag NPs on ITO BRs is larger than that on the ITO film, increasing the *θ* and dewetting of Ag layer from the ITO BRs.

### Thermal annealing for enhanced dewetting and agglomeration

To clarify the dewetting behavior of Ag NPs, thermal annealing was conducted at various temperatures because thermal energy helps the Ag atoms to find thermodynamically stable sites. At 100 °C, the Ag layer on ITO BRs was more dewetted, resulting in spherical Ag NPs (Fig. 1b). This temperature is relatively low compared to the temperature required for the dewetting of the Ag NPs on the ITO thin-film (200 °C) (Figure S1). The low-temperature dewetting of the Ag NPs on ITO BRs originated from the small size of Ag nuclei. The small particle can agglomerate easier than the bulk Ag layer because the melting point of the nanodot becomes lower as the size gets smaller^56,57^. When the annealing temperature increased from 100 to 300 °C, the averaged size (D_avg_) of the Ag NPs on ITO BRs gradually increased from 25 to 37 nm due to agglomeration (Fig. 1c). During the agglomeration, Ag atoms can diffuse along the surface of ITO BR. So, the shape of Ag NPs was changed from sphere to ellipsoid. In the case of Ag NPs on ITO film, the average size of Ag NPs was 81 nm at 200 °C and increased to 109 nm at 300 °C. It is notable that the Ag NPs formed on the ITO BRs are small, have high density and spherical shape. But, the Ag NPs on ITO films are large, have low density and hemispherical shape.

**Figure 2:**
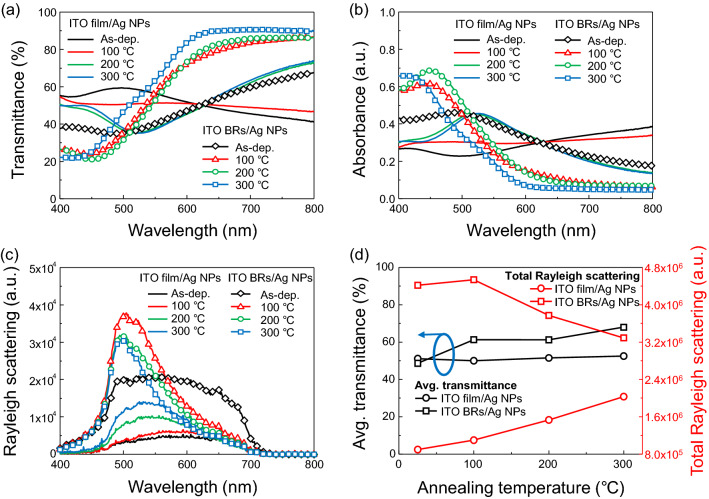
Optical properties of Ag NPs on ITO film and ITO BRs with thermal annealing temperature. (**a**) Optical transmittance, (**b**) absorbance and (**c**) Rayleigh scattering spectra of Ag NPs on ITO film and ITO BRs. Optical properties were measured on samples that were not heat-treated and samples annealed at 100, 200, and 300 °C. (**d**) Average optical transmittance (400 ≤ λ ≤ 800 nm) and total Rayleigh scattering plotted as a function of annealing temperature. The total Rayleigh scattering was determined by integrating each resonance spectrum over the range of 400 ≤ λ ≤ 800 nm.

Optical transmittance (*OT*) of Ag NPs formed on ITO film and ITO BRs was measured for as-deposited Ag (10 nm) and thermally annealed samples at 100 °C, 200 °C and 300 °C (Fig. 2a). The ITO film/Ag NPs films showed average *OT* = 51.1% at room temperature, *OT* = 50.0% at 100 °C, *OT* = 51.5% at 200 °C and *OT* = 52.5% at 300 °C. In the case of as-deposited Ag NPs on ITO BRs, the average *OT* was as low as 48.6%. But, as the annealing temperature increased, *OT* gradually increased to 61.2% at 100 °C, *OT* = 61.2% at 200 °C and *OT* = 68.0% at 300 °C. To observe the plasmonic light absorption by Ag NPs, absorbance spectra were plotted as a function of wavelength (Fig. 2b). No strong absorption peak was observed in the as-deposited Ag layer on the ITO film, but, the thermally annealed samples exhibited a plasmonic absorption peak at λ = 530 nm. The ITO BRs/Ag NPs showed stronger intensity and narrower absorbance spectrum than ITO film/Ag NPs because of LSPs. It is worth to note that the absorbance peak of ITO BRs/Ag NPs shifted from 460 to 410 nm as the annealing temperature increased from 100 to 300 °C. This peak shift could be due to changes in the size and shape from sphere to ellipsoid.

Because the intensity of the dark-field Rayleigh scattering is an easy way to know the coupling of surface plasmons at the Ag NPs, dark-field Rayleigh scattering spectra were measured to observe the effect of annealing temperature on the plasmonic light scattering (Fig. 2c). ITO film/Ag NPs showed negligible scattering intensity at 100 °C. But, when the Ag layer was annealed at 200 °C, the scattering intensity drastically increased and a plasmonic peak appeared at λ = 540 nm due to the formation of hemispherical Ag NPs. In the case of ITO BRs/Ag NPs, the scattering intensity was much higher than ITO film/Ag NPs due to both light scattering by ITO BRs and plasmonic light scattering by Ag NPs. After the thermal annealing at 100 °C, the scattering intensity further increased on plasmonic wavelengths (470 ≤ λ ≤ 550 nm). The narrow and strong scattering spectrum indicates strong plasmonic light scattering.

To find the optimum annealing temperature, average *OT* and total Rayleigh scattering intensity were plotted as a function of annealing temperature (Fig. 2d). The total Rayleigh scattering intensity was determined by integrating each resonance spectrum over the range of 400 ≤ λ ≤ 800 nm. As the annealing temperature increased, the average *OT* of ITO film/Ag NPs remained nearly constant and the total scattering intensity gradually increased. In the case of ITO BRs/Ag NPs, *OT* increased*,* but the total scattering intensity decreased. The maximum light scattering by ITO BRs/Ag NPs was obtained at 100 °C. For the efficient light trapping in optoelectronic devices, both high *OT* and strong light scattering are required. So, we chose the annealing temperature of 300 °C for ITO film/Ag NPs and 100 °C for ITO BRs/Ag NPs. Also, the size of Ag NPs can be tuned by increasing the thickness of the Ag layer. The relationship between the thickness of the Ag layer and the size of Ag NPs was experimentally demonstrated (Figure S2–S4 and Table S1) and it was found that 10 nm was the optimum thickness.

### Microstructure and chemical composition of ITO BRs/Ag NPs

**Figure 3:**
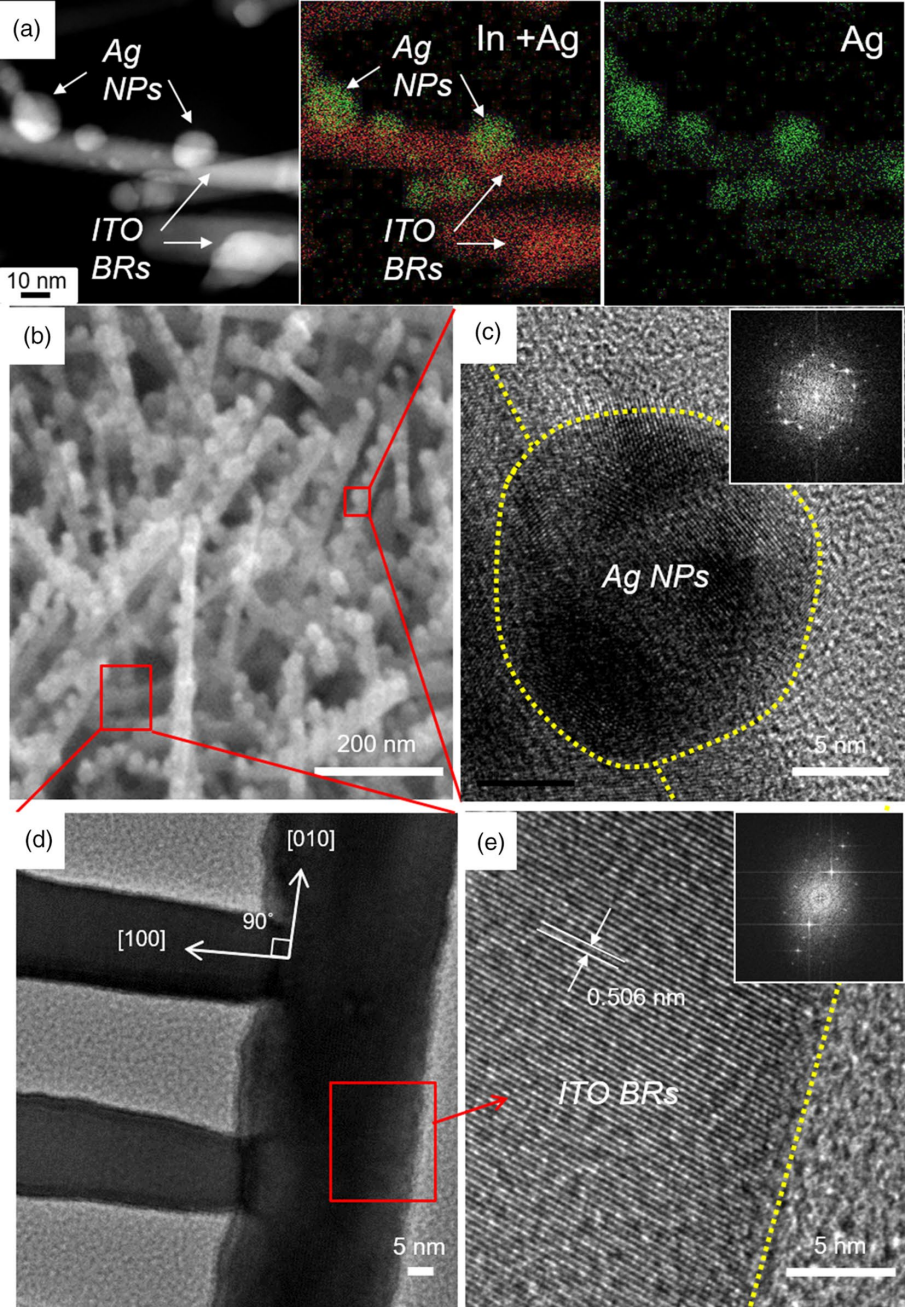
Analysis of microstructure of ITO BRs/Ag NPs. (**a**) STEM image and TEM-EDS map of indium (In) and silver (Ag). Red dots: In and green dots: Ag. (**b**) High-magnification SEM image of ITO BRs/Ag NPs. HR-TEM image of the (**c**) Ag NPs and (**d**, **e**) ITO BRs. The ITO BRs had branches on the sidewall with 90° rotation angle. The insets are the selected area electron diffraction patterns, indicating face-centered cubic structure of Ag NP and single-crystalline ITO with (100) orientation.

To verify the atomic compositions of ITO BRs and Ag NPs, energy-dispersive X-ray spectroscopy mapping was conducted (Fig. 3a). A red contour represents the atomic distribution of indium (In) and green indicates the Ag atoms. The red dots uniformly distributed along the ITO BRs and the green dots segregated on the Ag NPs. Although Ag atoms showed a bright contrast at NPs, a little amount of Ag atoms remained on the surface of ITO BRs. This result indicates that Ag atoms diffused along the surface of ITO BRs, leaving a few monolayers of Ag atoms on ITO BRs. High-magnification SEM image showed that the ITO BRs were randomly grown on the substrate and the Ag NPs were attached on the sidewalls of ITO BRs (Fig. 3b). Ag NPs had spherical shape and polycrystalline face-centered cubic structure (Fig. 3c). In the junction between the ITO BRs (Fig. 3d), a continuous atomic arrangement could be confirmed with the {100} family plane orientation of cubic-based bixbyite In_2_O_3_ having d-spacing of 0.506 nm (Fig. 3e). Because the bixbyite In_2_O_3_ {100} family planes such as (100), (010), and (001) have 90° rotation angle^58^, the ITO BRs are perpendicular to each other. We further conducted an X-ray diffraction (XRD) analysis to confirm the orientations of ITO and Ag NPs. ITO films have the polycrystalline orientation as evidenced by (211), (222), (400), (411), (431), (440) and (622) planes (Figure S5). In the case of Ag NPs (10 nm), the size of crystallite was too small to be detected by the XRD spectrometer used in this study. Despite ITO BRs have (100) single crystalline structure, various orientations originated from the polycrystalline ITO layer beneath the ITO BRs were observed in the XRD patterns.

**Figure 4:**
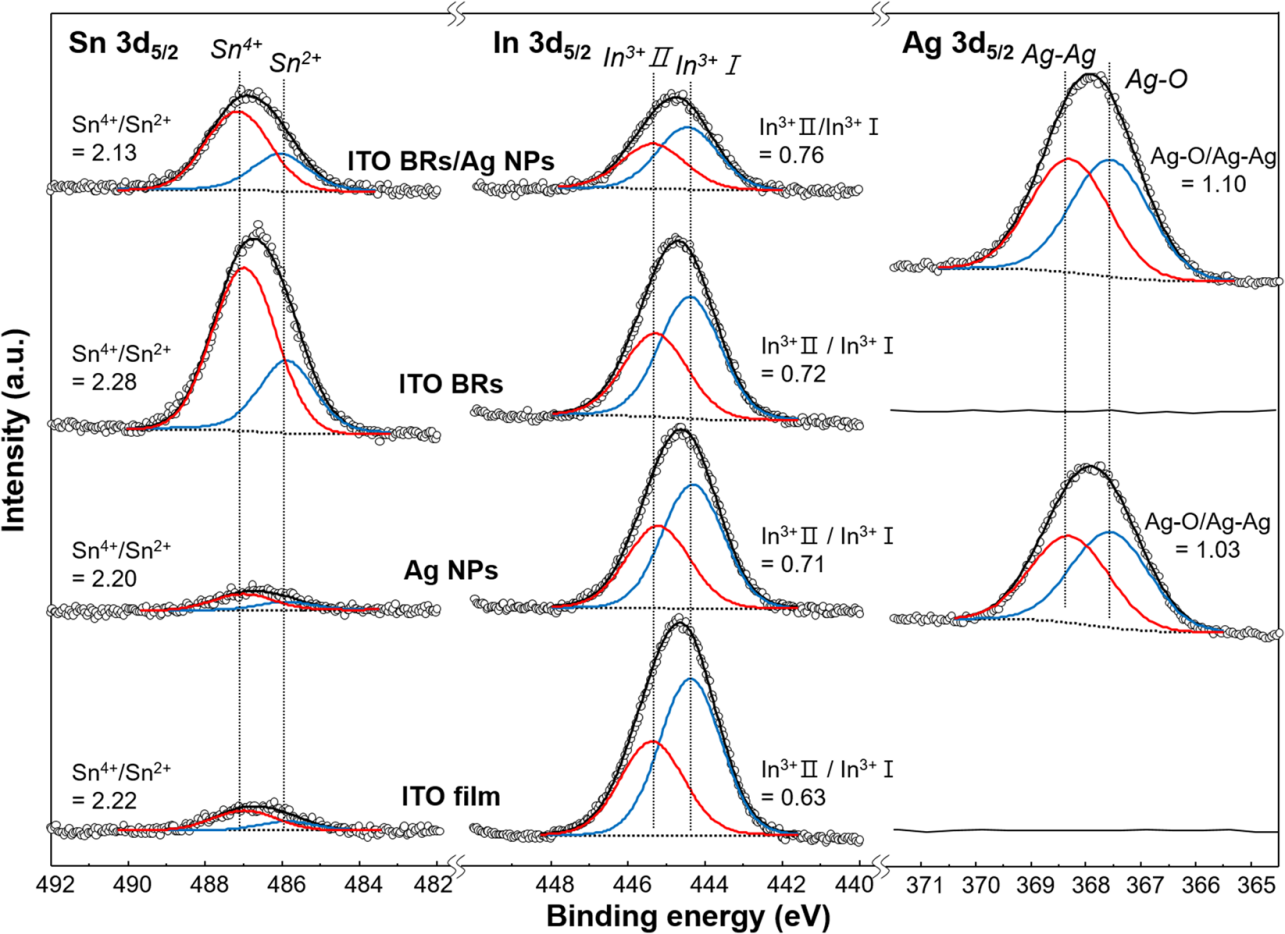
Chemical composition and bonding state. Sn 3*d*_5/2_, In 3*d*_5/2_, Ag 3*d*_5/2_ X-ray photoelectron spectroscopy (XPS) spectra of ITO film, Ag NPs, ITO BRs and ITO BRs/Ag NPs.

In order to investigate the bonding states and chemical compositions of ITO film, Ag NPs, ITO BRs and ITO BRs/Ag NPs, the deconvolution of Sn 3*d*_5/2_, In 3*d*_5/2_, Ag 3*d*_5/2_, and O 1*s* core-level was carried out (Fig. 4). The Sn 3*d*_5/2_ XPS spectra were fitted into Sn^2+^ at 486.1 eV, and Sn^4+^ at 487.2 eV^59^. Although all the samples showed a similar ratio of Sn^4+^/Sn^2+^ (2.15–2.26), the intensity of Sn 3*d*_5/2_ spectra was higher on ITO BRs and ITO BRs/Ag NPs than on ITO film and Ag NPs, meaning that the ITO BRs intrinsically have a large amount of Sn. The high content of Sn might be originated from Sn catalysts formed at the end of ITO BRs during the vapor–liquid–solid growth^58^. The In 3*d*_5/2_ spectra were fitted with two bonding states of In^3+^I (444.4 eV) and In^3+^II (445.4 eV)^60^. The two bonding states of In^3+^ originated from a screening effect caused by Sn doping: the screened (In^3+^I) and unscreened (In^3+^II) states. The intensity ratios of In^3+^II to In^3+^I were 0.76 for ITO film, and 0.72 for ITO BRs. According to the previous work, the increase in the unscreened states of In^3+^ is mainly due to a decrease in the surface carrier concentration, which increases the workfunction of ITO^61^. The presence of Ag NPs was confirmed by the detection of Ag 3*d*_5/2_ spectra in Ag NPs and ITO BRs/Ag NPs. The ratio of Ag–Ag bond (368.3 eV) and Ag–O bond (367.5 eV) have a similar value of ~ 1, meaning that the surface of Ag NPs was partially oxidized during the vacuum heat treatment process for dewetting the Ag layer. The peak for O 1*s* was fitted with two components at binding energies of 529.3 eV and 530.8 eV (Figure S6). The peak at 530.4 eV was identified to be because of O–M bond and the peak centered at 531.8 eV could be assigned to superimposed three components of O–H, O–C, and (O_2_)^2−^^62^. The intensity of O^2−^ peak increased with coating of Ag NPs on ITO film and ITO BRs, which indicates enrichment of oxygen atoms during the thermal annealing of Ag NPs.

### Optical properties of ITO BRs/Ag NPs

**Figure 5:**
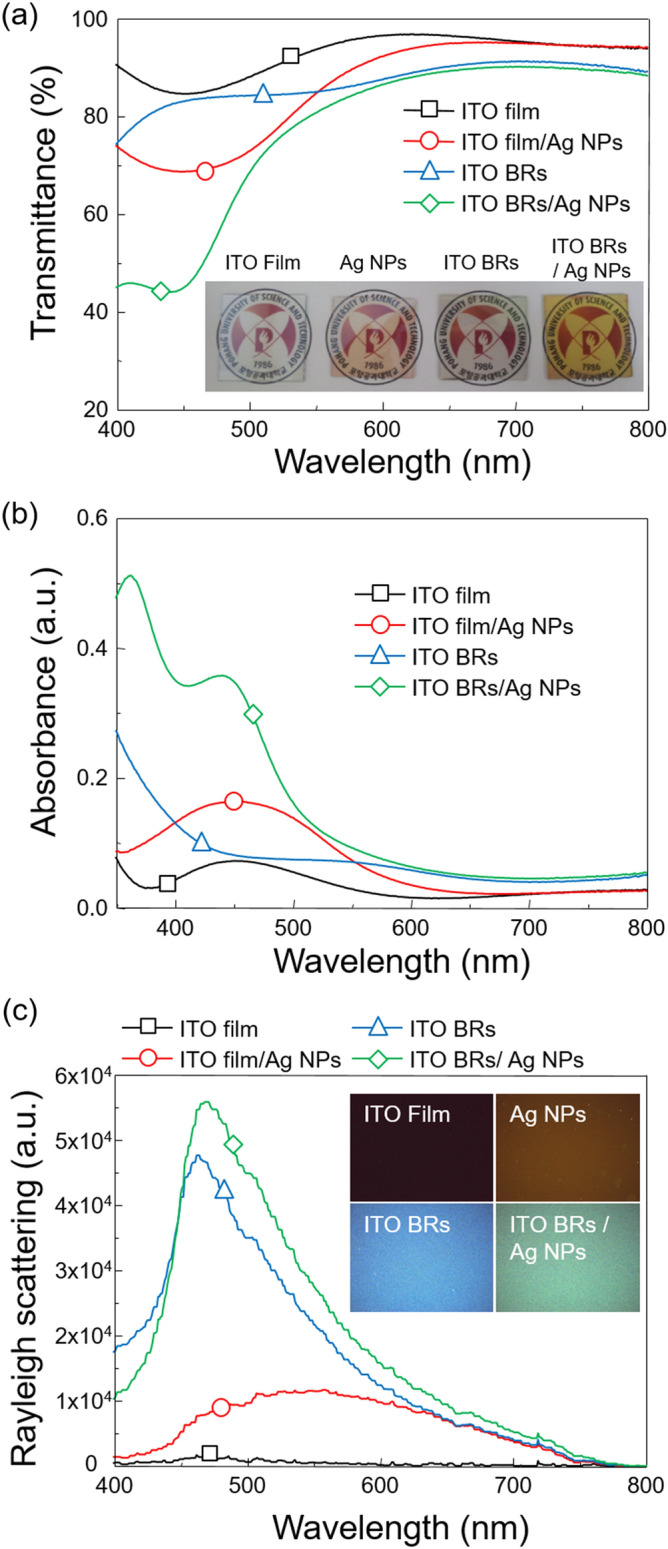
Optical properties of ITO film, ITO film/Ag NPs, ITO BRs and ITO BRs/Ag NPs. (**a**) Optical transmittance of ITO film, Ag NPs, ITO BRs, and ITO BRs/Ag NPs. Inset: photographs of samples prepared on the glass substrates. (**b**) UV–visible absorbance spectra. (**c**) Dark-field Rayleigh scattering spectra measured by dark-field microscopy with transmission mode. Insets: dark-field images.

Optical transmittance (*OT*) of four-different samples of ITO film, Ag NPs, ITO BRs, and ITO BRs/Ag NPs was measured as a function of wavelength (Fig. 5a). Although *OT* of Ag NPs, ITO BRs and ITO BRs/Ag NPs were lower than that of the ITO film, there was no severe loss in *OT* on the wavelengths (500 ≤ λ ≤ 800 nm). In absorbance spectra (Fig. 5b), a characteristic peak of the plasmonic extinction of Ag NPs was observed in a specific wavelength region (400 ≤ λ ≤ 500 nm). The ITO film/Ag NPs showed relatively lower peak intensity and broader shape of the absorbance spectrum than ITO BRs/Ag NPs. The narrow and intensive absorbance spectrum of ITO BRs/Ag NPs implies strong plasmonic light interaction. To measure the amount of scattered light by nanostructures, we measured dark-field Rayleigh scattering spectra using dark-field microscopy (Fig. 5c). In ITO BRs/Ag NPs, a synergistic effect of plasmon coupling by Ag NPs and light scattering by ITO BRs results in the strongest scattering intensity. The ITO BRs showed the second strongest scattering intensity because ITO BRs effectively scatters the incident light to broad angles. The Ag NPs showed small peak intensity over wavelength ranges from (450 ≤ λ ≤ 750 nm) and the ITO film did not exhibit any scattering behavior. To investigate the existence of surface plasmon coupling in detail, we captured dark-field images (Inset). The ITO BRs/Ag NPs showed the brightest green color which originated from the combination of surface plasmon coupling in Ag NPs and light scattering by ITO BRs. The ITO BRs exhibited blue images because of photon scattering at short wavelength region (400 ≤ λ ≤ 500 nm). The dark brown color was obtained in Ag NPs and the ITO film presented a black image.

### Application in organic solar cell

**Figure 6:**
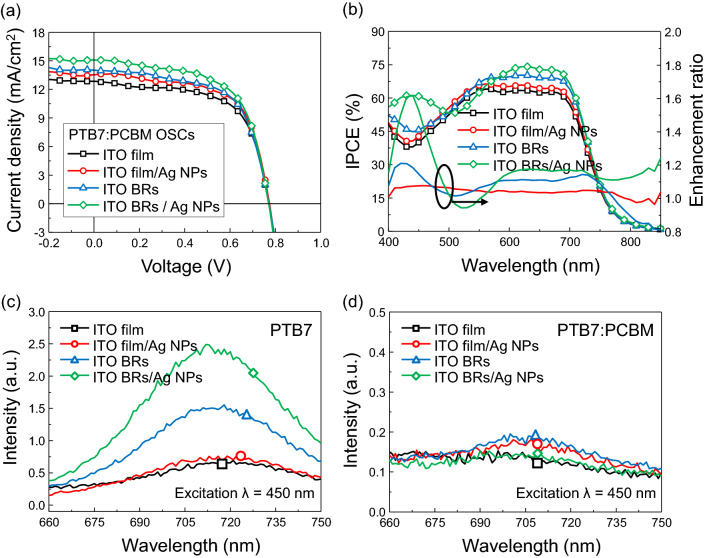
Charaterization of organic solar cells. (**a**) J–V characteristics and (**b**) incident photon to current conversion efficiency (IPCE) of PTB7:PCBM OSCs fabricated on ITO film, Ag NPs, ITO BRs and ITO BRs/Ag NPs. Steady-state photoluminescence spectra of (**c**) PTB7 films and (**d**) PTB7:PCBM layers.

To evidence, the effect of 3D plasmonic nanostructures on efficiency enhancement of OSCs, PTB7:PCBM OSCs were fabricated. The OSCs consisted of ITO (170 nm)-coated glass substrate, PEDOT:PSS (40 nm) hole transport layer, PTB7:PCBM (100 nm) active layer, bathocuproine (BCP) (15 nm) cathode interlayer and Ag (120 nm) reflector (Figure S7). The cells on the ITO film exhibited open-circuit voltage (V_oc_) = 0.78 V, short-circuit current (J_sc_) = 13.0 mA/cm^2^, fill factor (FF) = 62.6%, and average PCE = 6.3% (Fig. 6a; Table 1). When the Ag NPs were formed on the ITO film, J_sc_ increased to 13.5 mA/cm^2^ and the average PCE increased to 6.6%. This increase in efficiency stands for the enhancement of light scattering by plasmonic light coupling. The OSCs with ITO BRs also increased J_sc_ = 14.1 mA/cm^2^ and PCE = 6.6% because of enhanced charge carrier transport and light scattering. When the devices were fabricated on the ITO BRs/Ag NPs, the highest average PCE = 7.1% was achieved. To find the reason for the improved photocurrent, we measured incident photon-to-current conversion efficiency (IPCE) (Fig. 6b). The devices with Ag NPs, ITO BRs, and ITO BRs/Ag NPs showed photocurrent enhancement on the broadband wavelengths (400 ≤ λ ≤ 800 nm). In particular, the ITO BRs/Ag NPs exhibited strong enhancement on the plasmonic wavelength region (400 ≤ λ ≤ 500 nm). Even though the ITO BRs effectively scattered photons (Fig. 5c), the enhancement ratio was smaller than that of ITO BRs/Ag NPs because there were no LSPs from plasmonic NPs. These results evidence that not only the light scattering but also, the electric field enhancement by LSPs played a key role in enhancing the photocurrent in OSCs.

**Table 1:** Photovoltaic characteristics of the devices.

Electrodes	V_oc_ (V)	J_sc_ (mA/cm^2^)	FF (%)	PCE (%)
ITO film	0.78	13.0	62.6	6.3 ± 0.2
Ag NPs	0.78	13.5	63.1	6.6 ± 0.2
ITO BRs	0.78	14.1	61.6	6.8 ± 0.1
ITO BRs/Ag NPs	0.78	14.8	61.5	7.1 ± 0.1

The illumination condition used for the measurements was AM 1.5 G 100 mW/cm^2^.

Influence of Ag NPs, ITO BRs and ITO BRs/Ag NPs on optoelectronic properties of PTB7 (Fig. 6c) and PTB7:PCBM (Fig. 6d) was investigated by steady-state photoluminescence (PL) measurements. The PTB7 films with ITO BRs showed higher PL intensity than ITO film and Ag NPs, meaning that ITO BRs contribute to the light absorption and the exciton generation in PTB7. It is also notable that the PL intensity is considerably higher in PTB7 film with ITO BRs/Ag NPs than that with ITO BRs because ITO BRs/Ag NPs form a strong localized electric field and increase exciton generation in the active layer. The PL from the electron donor (PTB7) is known to be quenched by addition of electron acceptor (PC_70_BM) because electrons in lowest unoccupied molecular orbital (LUMO) level of PTB7 spontaneously transfer to LUMO level of PC_70_BM^63^. Therefore, PL intensities were completely quenched in all samples after the addition of PC_70_BM (Fig. 6d). This means that the electrons in PTB7 transfer to PC_70_BM and generate photocurrent in PSCs. A large amount of exciton generation and effective quenching of PL in ITO BRs/Ag NPs verifies the enhanced photocurrent of OSCs.

In order to confirm other factors that might improve the photovoltaic performance, we measured the surface energy of four transparent electrodes of ITO film, Ag NPs, ITO BRs, and ITO BRs/Ag NPs by measuring the contact angle of water and diiodomethane (Figure S8). Then, the effect of surface energy on the charge carrier mobility of PTB7:PCBM layer was investigated by measuring the space-charge limited current (Figure S9). Despite the total surface energy was a little larger on ITO BRs (74.9 mJ/m^2^) and ITO BRs/Ag NPs (74.8 mJ/m^2^) than ITO film (63.8 mJ/m^2^) and ITO Ag NPs (60.7 mJ/m^2^), all the hole-only devices in configuration of electrode/PEDOT:PSS/PTB7:PCBM/Au showed nearly similar hole mobility regardless of the type of electrodes, indicating that the surface energy of electrodes does not change the charge carrier mobility of PTB7:PCBM active layer. But, the trends in current density (ITO BRs/Ag NPs ≈ ITO BRs > Ag NPs > ITO film) evidences that the increased surface roughness by Ag NPs and ITO BRs improved the electrical properties by reducing the resistance between the electrodes and PTB7:PCBM layers. We further conducted experiments to demonstrate whether the surface energy of electrodes affects the adhesive strength of PTB7:PCBM active layer on the electrodes (Figure S10). It is noticeable that PTB7:PCBM layer was peeled off from the ITO film and transferred to the adhesive tape. But, PTB7:PCBM layers coated on Ag NPs, ITO BRs and ITO BRs/Ag NPs electrodes were not peeled off from the substrate. Even the adhesive glue was transferred from tape to ITO BRs and ITO BRs/Ag NPs. From these results, we found that the physical adhesion between the electrode and the polymer layer is more dependent on nanostructures in between the electrode and active layer rather than the surface energy. The nanostructures between the electrodes and the polymers are expected not only to improve adhesion but also to improve electrical properties in some extent by increasing the contact area between the electrode and the active layer.

### Optical simulation

**Figure 7:**
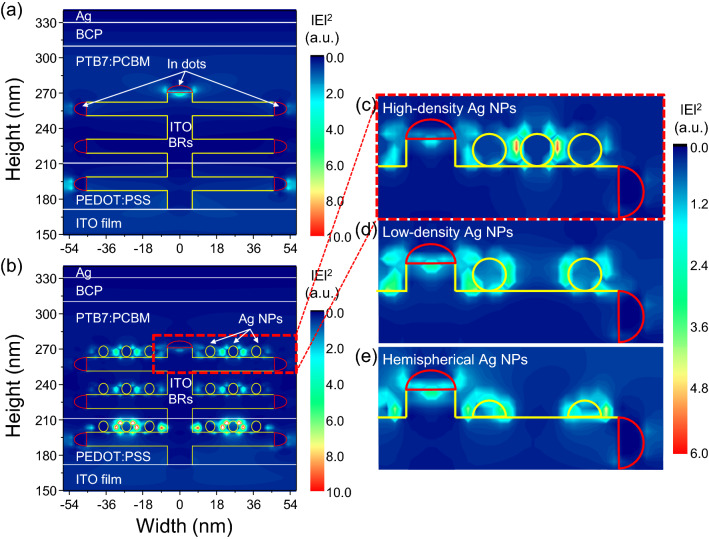
Optical simulation of ITO BRs/Ag NPs in organic solar cells. (**a**) Simulated cross-sectional electric field distribution in PTB7:PCBM OSCs with (**a**) ITO BRs and (**b**) ITO BRs/Ag NPs. Transverse magnetic waves were illuminated from the surface normal direction at a wavelength of 450 nm. Yellow spheres are Ag NPs and red hemispherical dots at the ends of ITO BRs are metallic In catalysts. Electric field distribution near the Ag NPs having (**c**) high-density and spherical shape, (**d**) low-density and spherical shape and (**e**) low-density and hemispherical shape.

We analyzed the plasmonic light coupling in ITO BRs/Ag NPs using finite-domain time-difference (FDTD) optical simulation (Fig. 6 and S11). The main objective is to understand the effect of near-field enhancement in Ag NPs embedded into the interfacial layer (PEDOT:PSS) and photoactive layer (PTB7:PCBM). For the ITO BRs, we assumed a height of 100 nm, a branch length of 40 nm, and a diameter of 12 nm. Because plasmonic light trapping is related to the transverse magnetic (TM) waves, results from TM waves were presented at the plasmonic wavelength of 450 nm. The ITO BRs showed any electric field enhancement, but a small distortion of propagation direction was observed due to light scattering (Fig. 7a). In contrast, strong electric field enhancement was found near the Ag NPs on ITO BRs (Fig. 7b). The electric field from the Ag NPs can propagate ~ 10 nm. Therefore, near-field enhancement from Ag NPs embedded into PEDOT:PSS layer cannot contribute to the generation of photoelectron in PTB7:PCBM. Only the Ag NPs in the active layer could contributes the photocurrent enhancement in OSCs. In order to observe the geometric effects of Ag NPs, we changed the density and shape of Ag NPs (Fig. 7c–e). In the case of high-density Ag NPs, the electric field between Ag NPs was extremely strong (Fig. 7c). This strong electric field, called hot-spot, was originated from the interaction between surface plasmons on two neighboring Ag NPs. The hot-spot can only be obtained if the distance between Ag NPs is less than twice the propagation length (~ 20 nm). When the Ag NPs were loosely packed, the hot-spot disappeared, resulting in reduced light absorption in the active layer (Fig. 7d). In the case of hemispherical Ag NPs, the electric field was focused at the corner of the hemisphere and a large amount of field was captured at the interface between ITO BRs and Ag NPs (Fig. 7e), leading to weak electric field in PTB7:PCBM layer. Based on these results, we can conclude that spherical Ag NPs attached to 3-dimensional ITO BRs provide great advantages over conventional low-density and hemispherical Ag NPs on ITO film.

## Discussion

Despite a little difference in workfunction (4.7–5.0 eV) of electrodes could be originated from their unique atomic compositions^60–62^, the hole extraction from PTB7:PCBM to transparent anodes facilitates regardless of electrodes because PEDOT:PSS hole transport layer with high workfunction (5.0 eV) is able to effectively align the energy level between ITO and HOMO of PTB7 (5.2 eV) (Figure S12). Also, the similar hole mobility of the PTB7:PCBM active layers fabricated on four different electrodes supports the fact that the composition and surface energy of the electrodes have little effect on the charge transport of PTB7:PCBM polymer solar cells. On the other hand, the IPCE measurements of polymer solar cells with ITO BRs/Ag NPs showed a significant enhancement of photocurrent on the plasmonic wavelength region (400 ≤ λ ≤ 500 nm). The steady-state PL measurements also provide evidence of enhanced light absorption and exciton generation in the PTB7 layer with ITO BRs/Ag NPs. The optical simulation exhibited that highly dense and spherical Ag NPs attached on ITO BRs produced stronger localized surface plasmons than conventional low-density and hemispherical Ag NPs on ITO film. From these results, we believe that the improvement of solar cell efficiency is mainly attributed to the enhanced light absorption by localized surface plasmons by Ag NPs, rather than by the improvement of electrical properties by other factors.

## Conclusion

In summary, we demonstrated the high-density spherical Ag NPs on the ITO BRs at low temperatures. The small Ag nuclei deposited on ITO BRs allowed them to be dewetted at low-temperature (100 °C) which is much lower than the temperature (200 °C) required for agglomeration of the Ag NPs on ITO film. The unique 3D structure of the ITO BRs induces additional line-tension in the deposited Ag NPs to increase the contact angle for spherical shape of NPs. The spherical Ag NPs built up the stronger localized surface plasmons than hemispherical Ag NPs formed on ITO film. Because the Ag NPs on ITO BRs were embedded in the active layer of the OSCs, electric field enhancement by the Ag NPs increased the light absorption and photocurrent. As a result, the PTB7:PCBM OSCs increased PCE from 6.3 to 7.1%. We expect this 3D plasmonic nanostructure can be extended to various applications such as plasmonic optical sensors and photodetectors.

## Methods

### Fabrication of Ag NPs and ITO nano-branches

ITO nano-branches (ITO BRs) were fabricated by electron beam evaporation method^58^. Tin doped (10%) indium oxide pellet (99.99%) was used as a source material. The deposition pressure was maintained at about 2 × 10^–4^ Torr and substrate temperature was held at 350 °C. ITO BRs were grown at a deposition rate of 1 nm/s, monitored by a quartz crystal. Following the growth of ITO BRs, Ag nanoparticles (Ag NPs) were formed by deposition of 10 nm-thick Ag layer on ITO BRs with electron beam evaporation and thermally annealed at various temperatures (100–300 °C ) for 1 min in a rapid thermal annealing system.

### Fabrication of organic solar cells

ITO (170 nm thick, ~ 10 Ω/sq) glass was used as the starting substrate. The ITO BRs/Ag NPs were deposited on the substrate using electron beam evaporation. Then, the samples were cleaned using UV-ozone treatment for 2 min with a power of 30 mW/cm^2^ and PEDOT:PSS was spin-coated and dried at 130 °C for 10 min. After PEDOT:PSS coating, the substrates were transferred to an N_2_-filled glove box (< 0.1 ppm O_2_ and H_2_O). PTB7 (purchased from 1-materials) was first dissolved in chlorobenzene, to make a 10 mg/ml solution, followed by blending with PC_70_BM, in a 1:1.5 weight ratio. The blend was spin-coated (1,600 rpm, 30 s) on top of the PEDOT:PSS. The devices were annealed on a hot plate in a glove box at 80 °C for 10 min. The active layer thickness was measured as ~ 100 nm by a surface profiler. The reflective cathode of BCP (15 nm)/Ag (120 nm) were deposited using thermal evaporation at a base pressure of 1 × 10^–6^ Torr.

### Characterizations

The current density–voltage curves were measured under air ambient using a Keithley 2,400 source meter. The photocurrent was measured under AM1.5G 100 mW/cm^2^ illumination from an Oriel 150 W solar simulator. The light intensity was calibrated using a mono-silicon detector by the National Renewable Energy Laboratory. The SEM was performed using a PHILIPS XL30S with an accelerating voltage of 10 kV and a working distance of 6 mm. The HR-TEM was done using a Cs-corrected JEM 2200FS operated at 200 kV. The optical transmittance and absorbance were measured using a UV–visible spectrometer (Agilent Technologies Cary 4000). The X-ray photoelectron spectroscopy (XPS) was measured using a 4D beam line, Pohang Accelerator Laboratory (PAL). X-ray diffraction (XRD) was performed on a Rigaku D/Max-2500 instrument. The incident photon to current conversion efficiency (IPCE) was measured using QEX10 Solar Cell Quantum Efficiency Measurement System (PV Measurements, Inc.) with 75 W xenon lamp fitted with a monochromator as a monochromatic light source.

### Simulation

Two-dimensional finite-domain time-difference (FDTD) simulation (Fullwave, Synopsys Inc.) was performed for studying the light absorption and electric field distribution in organic solar cells. The refractive indices and extinction coefficients of each layer were measured by ellipsometry. Boundary conditions for the FDTD simulation were set to be periodic in the horizontal direction and perfectly matched layer in the vertical direction to avoid unwanted reflection at the edge of the structure. The light was emitted by a plane wave on the surface normal direction. The source type was set to be a continuous wave. The grid size was 2 nm. The simulations were performed during the light propagation over a distance of 10 um in free space to reach steady-state. When the steady state was reached, the electric field distribution did not change over 0.1%. The cross-sectional discrete Fourier transformation monitor was used to obtain spatial electric field and absorption distributions at steady-state^64^.

## Supplementary information

Supplementary Information
