# Supplementary material for: Abnormal dewetting of Ag layer on three-dimensional ITO branches to form spatial plasmonic nanoparticles for organic solar cells
Source: Sci Rep. 2020 Jul 30;10:12819. doi: 10.1038/s41598-020-69320-4 (PMC7393491; doi:10.1038/s41598-020-69320-4)
Supplement: Supplementary file 1 — Supplementary Information [file 41598_2020_69320_MOESM1_ESM.pdf]

Supplementary Information

Abnormal dewetting of Ag layer on three-dimensional ITO branches to form spatial plasmonic nanoparticles for organic solar cells

Wan Jae Dong[1], Hak Ki Yu[2] and Jong-Lam Lee[1,]*

[1] Department of Materials Science and Engineering, Pohang University of Science and Technology (POSTECH), Pohang 790-784 (Republic of Korea)

[2] Department of Materials Science and Engineering & Deptartment of Energy Systems Research, Ajou University, Suwon, 16499 (Republic of Korea)

*Corresponding author: Department of Materials Science and Engineering, Pohang University of Science and Technology (POSTECH), Pohang, Republic of Korea. E-mail: jllee@postech.ac.kr.

Thermal annealing of Ag layer on ITO film

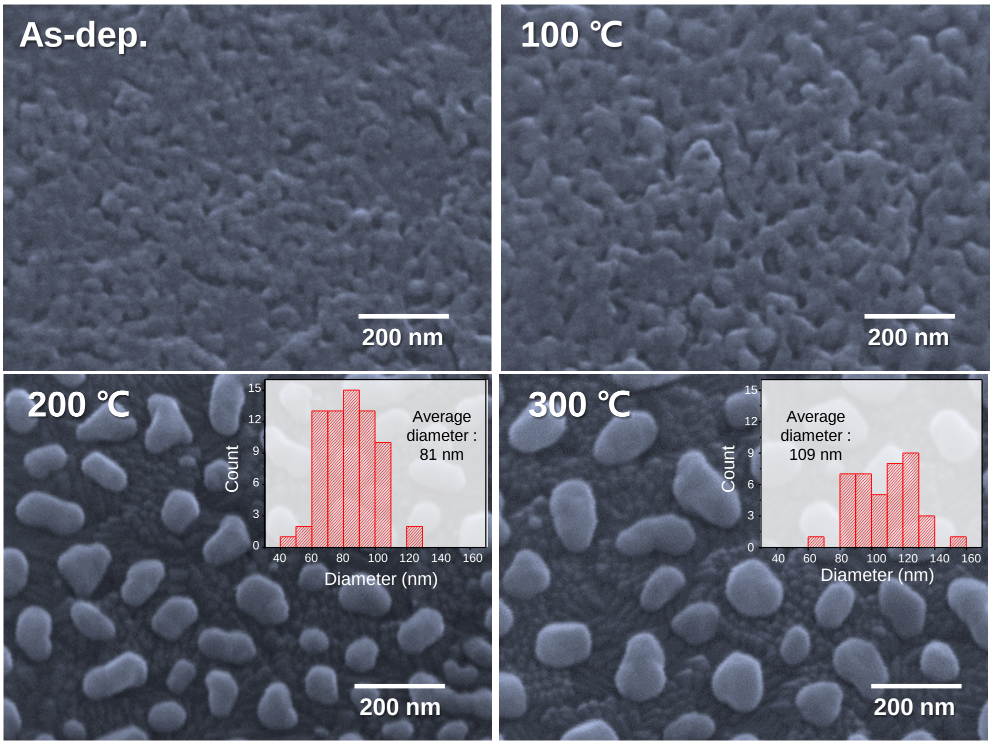

Figure S1. SEM images of as-deposited Ag layer (10 nm) on ITO film and thermally annealed samples at 100, 200 and 300 ˚C. Insets show the diameter of Ag NPs.

Thin Ag layer (10 nm) deposited on ITO film showed a network of islands (Figure S1). When the sample was thermally annealed at 100 ˚C, the shape of the island was more pronounced. The Ag layer began to aggregate at 200 ˚C, resulting in large Ag NPs having an average size of 81 nm. As the annealing temperature increased to 300 ˚C, the size of Ag NPs increased to 109 nm due to agglomeration.

Effect of Ag thickness on morphology, optical property and device performance

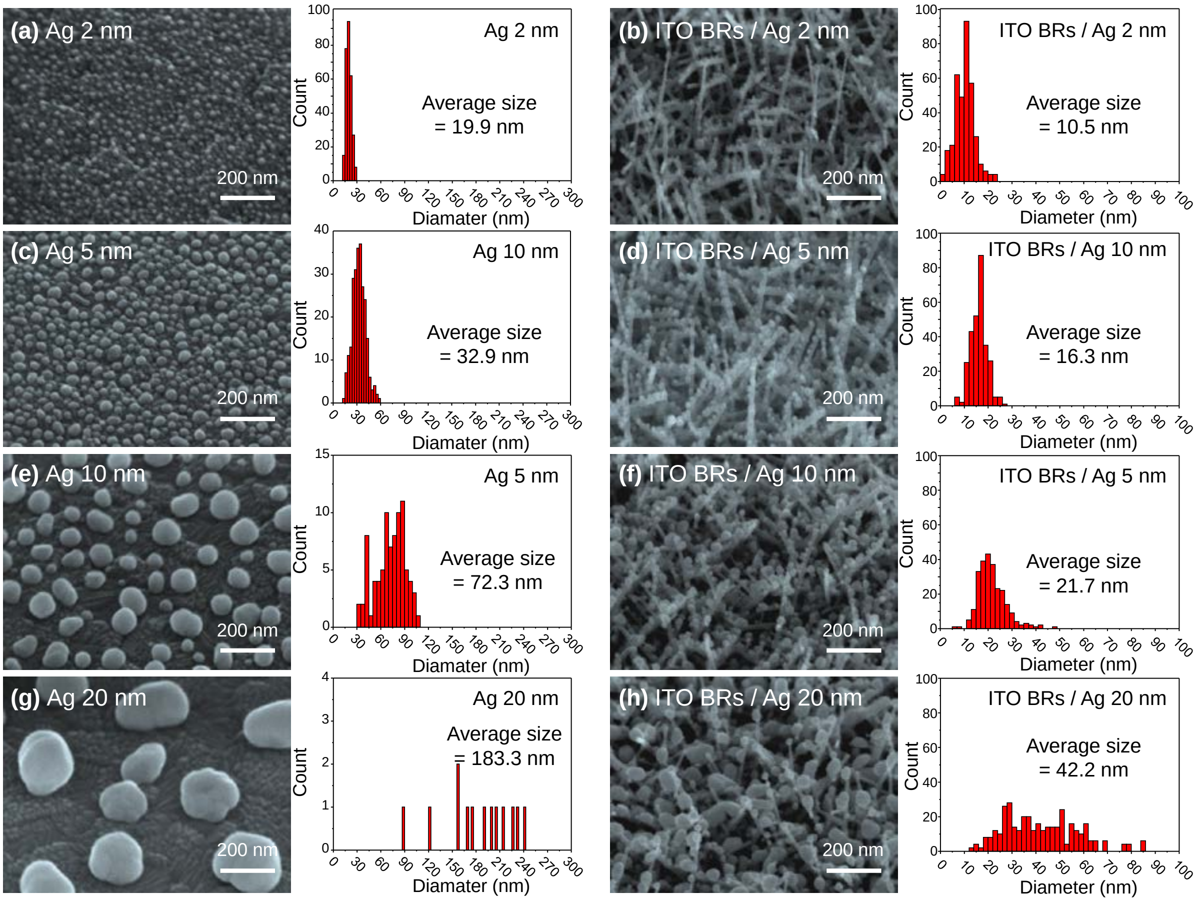

Figure S2. SEM images and size disbrituion of Ag NPs deposited on ITO film (left column) and ITO BRs (right column) for various Ag thickness of (a,b) 2 nm, (c,d) 5 nm, (e,f) 10 nm and (g,h) 20 nm.

Ag NPs were formed by deposition of Ag layer with thickness (t_{Ag}) of 2, 5, 10 and 20 nm and annealed at 300 ˚C for 1 min (Figure S2). As t_{Ag} increased from 2 to 20 nm, average diameter (D_{avg}) of Ag NPs formed on ITO film gradually increased while density of Ag NPs decreased. Ag NPs had D_{avg} = 19.9 nm at t_{Ag} = 2 nm, D_{avg} = 32.9 nm at t_{Ag} = 5 nm, D_{avg} = 72.3 nm at t_{Ag} = 10 nm, and D_{avg} = 183.3 nm at t_{Ag} = 20 nm. In case of Ag NPs formed on the ITO BRs, D_{avg} was smaller than the ones on the ITO films. The Ag NPs on ITO BRs had D_{avg} = 10.5 nm at t_{Ag} = 2 nm, D_{avg} = 16.3 nm at t_{Ag} = 5 nm, D_{avg} = 21.7 nm at t_{Ag} = 10 nm, and D_{avg} = 42.2 nm at t_{Ag} = 20 nm.

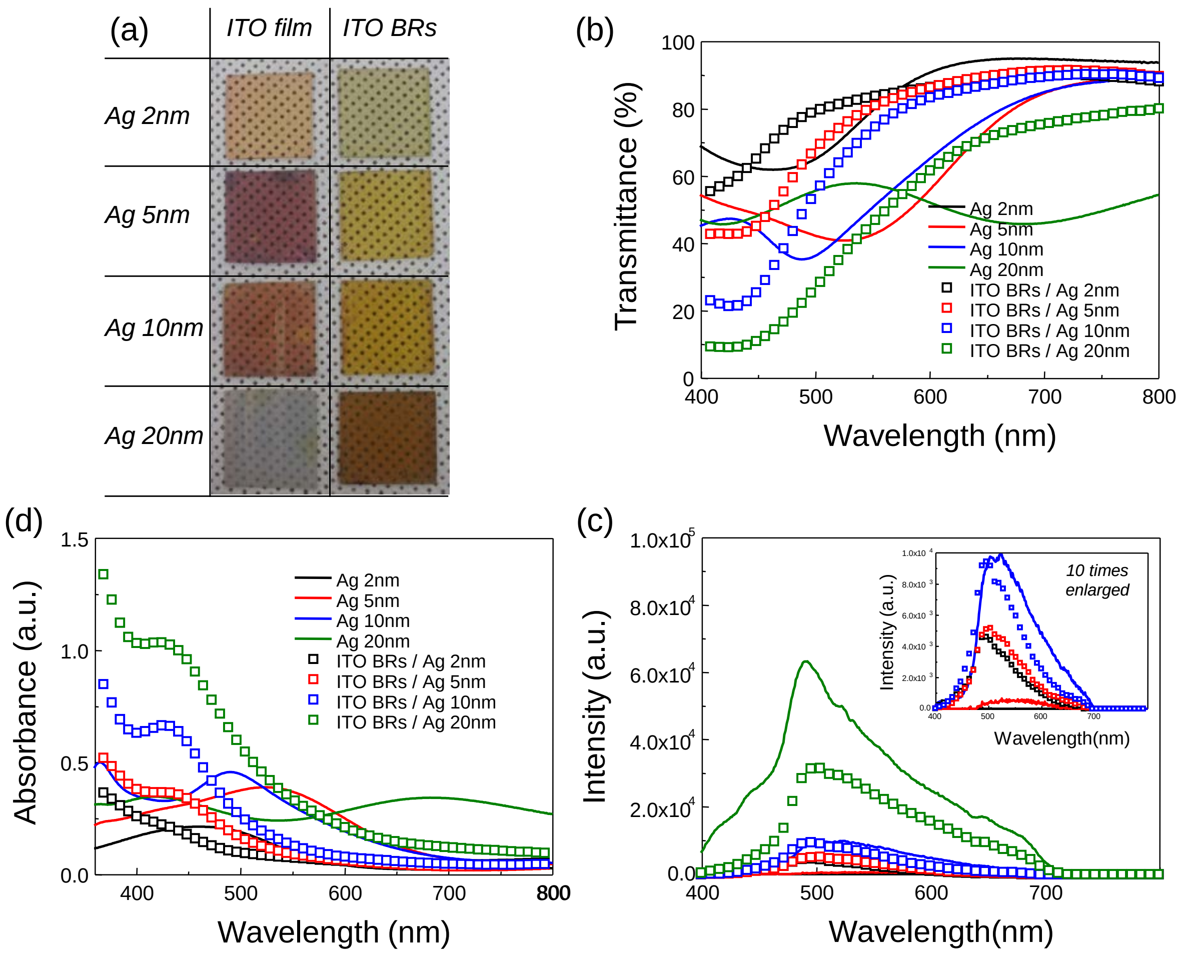

Figure S3. (a) Photographs of Ag NPs on ITO film and ITO BRs for various Ag thickness of 2, 5, 10 and 20 nm. (b) Optical transmittance, (b) absorbance and (c) dark-field Rayleigh scattering spectra plotted as a function of wavelength.

Photographs showed that the glass substrates covered with Ag NPs deposited on ITO films and ITO BRs (Figure S3a). As t_{Ag} increased on ITO films, colour of Ag NPs drastically changed to orange (t_{Ag} = 2 nm), purple (t_{Ag} = 5 nm), brown (t_{Ag} = 10 nm), and grey (t_{Ag} = 20 nm). On the other hand, the Ag NPs on ITO BRs showed a little change in color; bright yellow (t_{Ag} = 2 nm), yellow (t_{Ag} = 5 nm), orange (t_{Ag} = 10 nm), and brown (t_{Ag} = 20 nm). To find the reason for the color change, optical transmittance (*OT*) (Figure S3b), optical absorbance (*OA*) (Figure S3c), and dark-field Rayleigh scattering spectra (Figure S3d) were measured. Because Ag has an imaginary component to its refractive index, the *OT* decreased and *OA* increased as the t_{Ag} increased. It is notable that as the Ag thickness increased from 2 nm to 20 nm, *OA* peaks of Ag NPs on ITO films gradually shifted from 460 to 700 nm while no peak shift was observed in Ag NPs on ITO BRs (Figure S3c). *OA* peak of Ag NPs originated from plasmonic extinction which is closely related to size and inter-distance

between Ag NPs.[S1] Since size and inter-distance of Ag NPs on ITO BRs were not changed as much as those of Ag NPs on ITO film, OA peak did not shift and color did not significantly change. To quantify the amount of light scattering, we measured dark-field Rayleigh scattering (Figure S3d). Because the larger Ag NPs have a larger optical cross-section, the intensity of Rayleigh scatting increased as t_{Ag} increased from 2 to 10 nm.

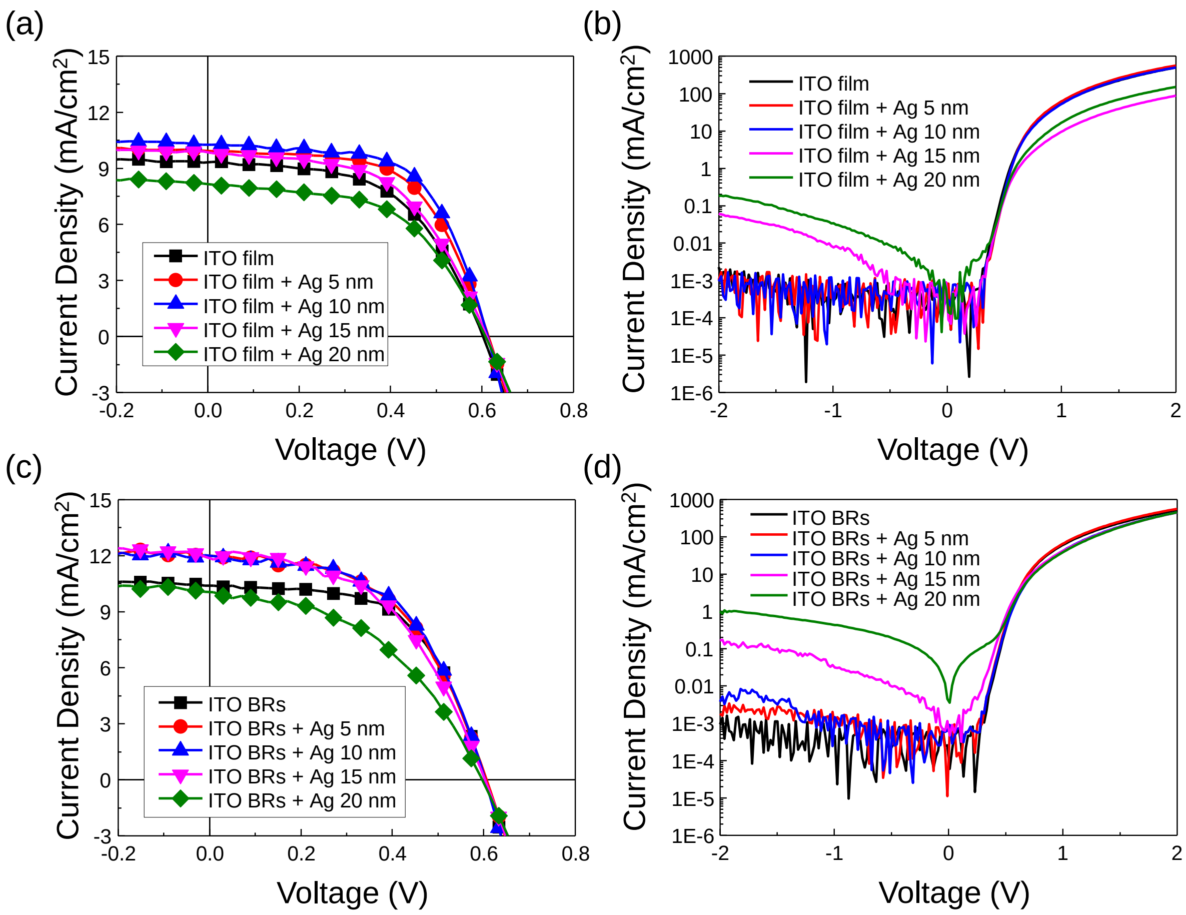

Figure S4. J-V characteristic of P3HT:PCBM bulk-heterojunction solar cells measured (a,c) under AM 1.5 G 100 mW/cm^2 light illumination and (c,d) under dark condition. The dark J-V curves were plotted in a logarithm scale. Various Ag NPs (0, 5, 10, 15 and 20 nm) were formed on (a,b) ITO film and (c,d) ITO BRs. The device structure is as follows: glass substrate / ITO film or ITO BRs / PEDOT:PSS (30 nm) / P3HT:PCBM (150 nm) / Bathocuproine (15 nm) / Al (100 nm).

To verify the size effect of Ag NPs on the performance of organic solar cells (OSCs), P3HT:PCBM bulk-heterojunction solar cells were fabricated and the J-V characteristics were measured (Figure S4). The device structure is as follows: glass substrate / ITO film or ITO

BRs / PEDOT:PSS (30 nm) / P3HT:PCBM (150 nm) / Bathocuproine (15 nm) / Al (100 nm). Among the devices with ITO film/Ag NPs, the maximum photo-conversion efficiency (PCE) of 3.70% was demonstrated with 10 nm-thick Ag NPs (summarized in Table S1). On the ITO BRs, the highest PCE = 3.89% was achieved with 10 nm-thick Ag NPs. The increase in PCE was mainly ascribed to increased J_{sc}. We believe that the increased J_{sc} originated from the plasmonic light trapping by Ag NPs. When the large Ag NPs (≥ 15 nm) were used in solar cells, PCE decreased due to increased dark-reverse current. The increase in roughness caused by Ag NPs might deteriorate the shunt resistance, resulting in leakage current. Therefore, optimum Ag thickness was found to be 10 nm.

Table S1. Photovoltaic characteristics of P3HT:PCBM solar cells. The illumination condition used for the measurements was AM 1.5 G 100 mW/cm^2.

**Substrate**	**V_{oc} (V)**	**J_{sc} (mA/cm^2)**	**FF (%)**	**PCE (%)**
ITO film	0.59	9.3	55.4	3.04
ITO film + Ag 5 nm	0.61	9.9	59.6	3.59
ITO film + Ag 10 nm	0.61	10.2	59.5	3.70
ITO film + Ag 15 nm	0.61	9.9	53.9	3.25
ITO film + Ag 20 nm	0.61	8.2	53.6	2.68
ITO BRs	0.61	10.4	57.2	3.63
ITO BRs + Ag 5 nm	0.61	12.0	51.7	3.78
ITO BRs + Ag 10 nm	0.61	12.0	53.1	3.89
ITO BRs + Ag 15 nm	0.59	11.9	51.7	3.63
ITO BRs + Ag 20 nm	0.59	10.1	46.6	2.78

Microstructure and chemical compositional analysis

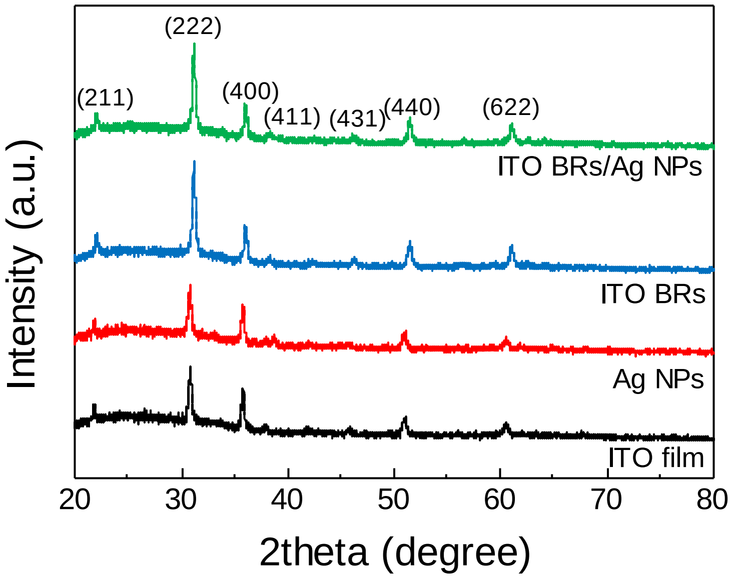

Figure S5. X-ray diffraction (XRD) patterns of ITO film, Ag NPs, ITO BRs, ITO BRs/Ag NPs. Only the XRD peaks of ITO were observed without detection of Ag peak.

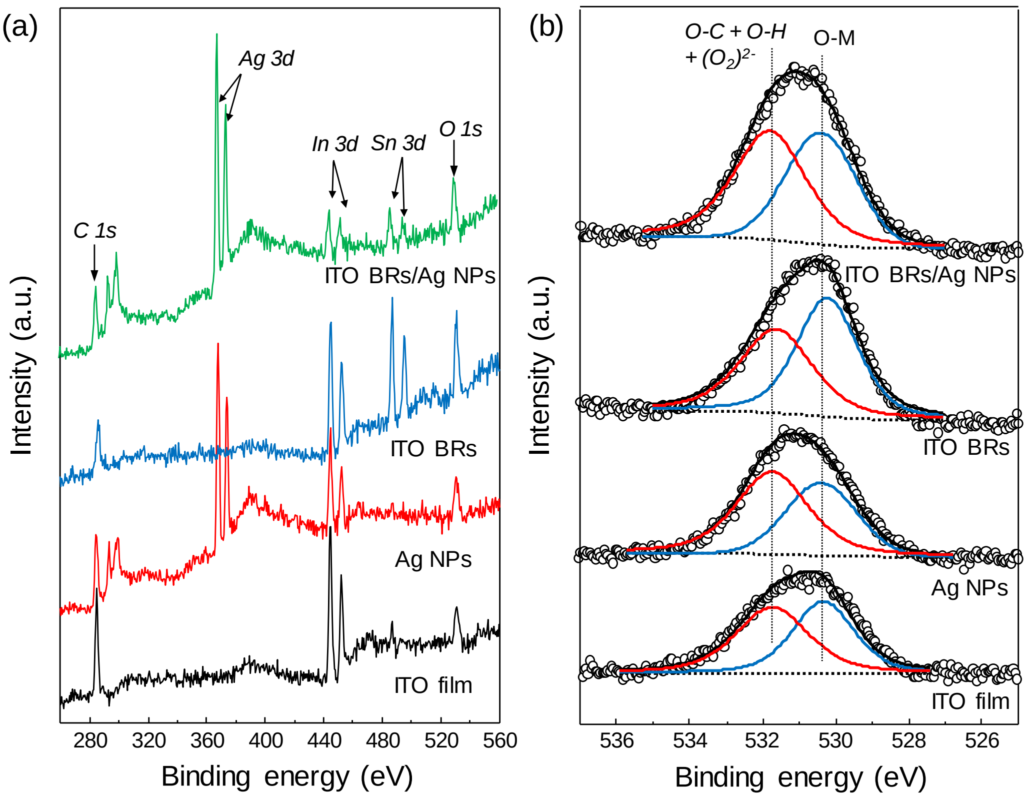

Figure S6. (a) Wide scan and (b) O 1s XPS spectra of ITO film, Ag NPs, ITO BRs, and ITO BRs/Ag NPs.

Device structure of PTB7:PCBM OSC fabricated on ITO BRs/Ag NPs

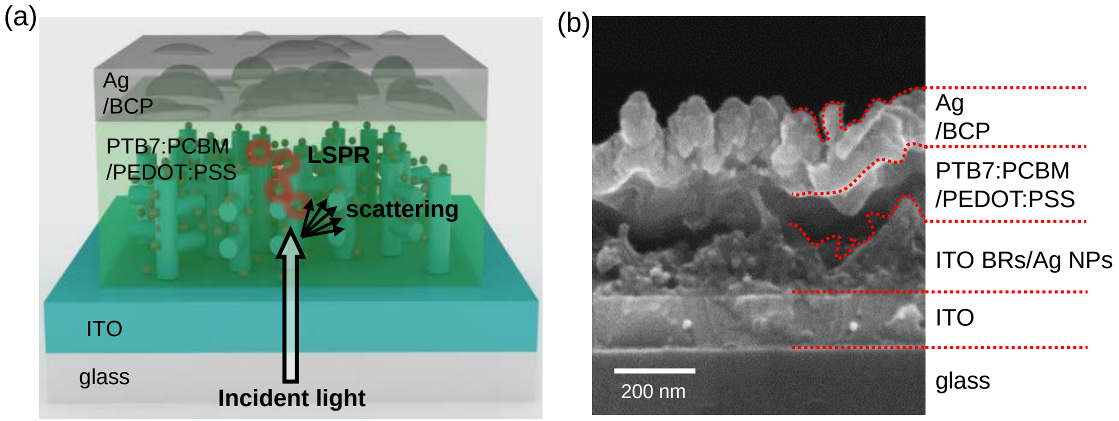

Figure S7. (a) Schematic illustration and (b) cross-sectional SEM image of PTB7:PCBM OSCs fabricated on ITO BRs/Ag NPs.

ITO BR/Ag NPs were conducted as the bottom electrode in PTB7:PCBM organic solar cells. The devices were consisted of ITO (170 nm)-coated glass substrate, ITO BRs/Ag NPs, PEDOT:PSS (40 nm) hole transport layer, PTB7:PCBM (80 nm) active layer, BCP (15 nm) cathode interlayer and Ag (120 nm) reflective electrode (Figure S7a). Cross-sectional SEM image (Figure S7b) showed that ITO BRs/Ag NPs are embedded into the active layer. Subsequently, the surface morphology of Ag/BCP electrode was roughened.

Effect of surface energy of electrodes on electronic properties and adhesion of PTB7:PCBM layer

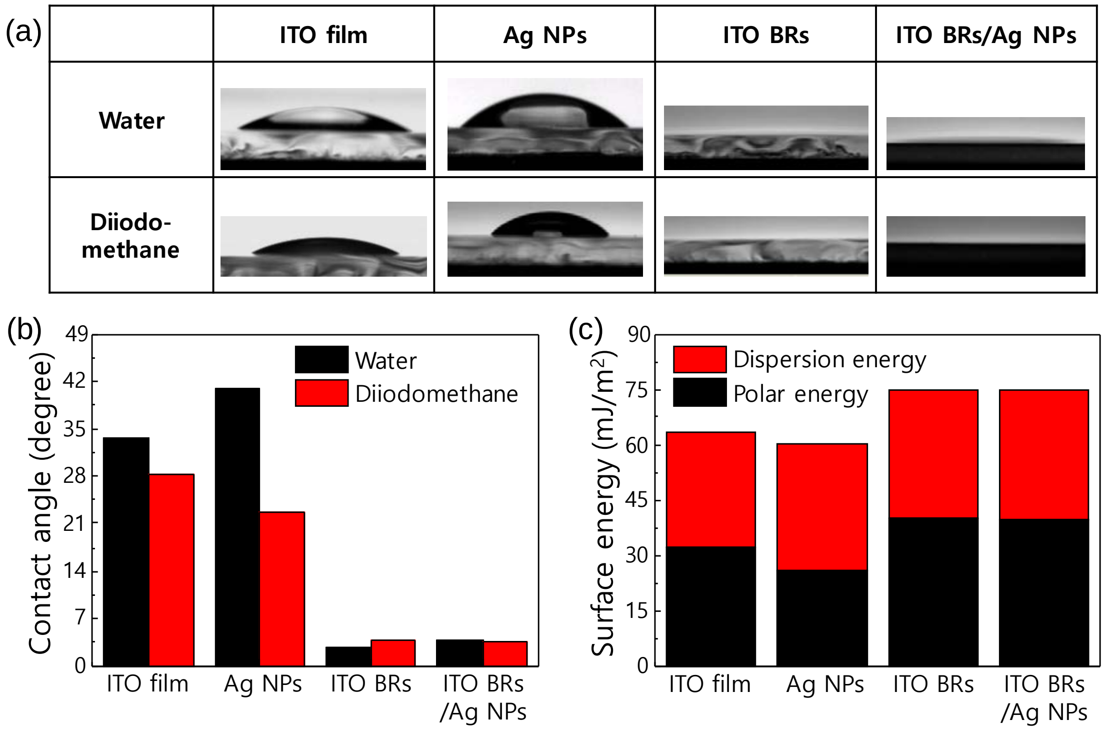

Figure S8. (a) Photographic images and (b) measured contact angle of water and diiodomethane droplets on ITO film, Ag NPs, ITO BRs and ITO BRs/Ag NPs. (c) Calculated polar and dispersion surface energies.

The contact angle made between the electrodes and droplets of water and diiodomethane was measured (Figure S8a). The water contact angle was 33.5°, 41.0°, 2.8°, 3.8°, and the diiodomethane contact angle was 28.3°, 22.5°, 3.8°, 3.6° for ITO film, Ag NPs, ITO BRs, and ITO BRs/Ag NPs, respectively (Figure S8b). It is noteworthy that ITO BRs and ITO BRs/Ag NPs have a much smaller contact angle of water and diiodomethane than ITO films and Ag NPs-coated ITO films due to the increased roughness factor of ITO BRs.[S2] From the measured contact angle, surface energies of electrodes were calculated. The following is a harmonic mean equation to get dispersive energy and polar energy:[S3]

$$(1+\cos\theta)\gamma_{pl} = 2(\gamma_s^d\gamma_{pl}^d)^{1/2} + 2(\gamma_s^p\gamma_{pl}^p)^{1/2}$$

where γ_s and γ_{pl} are the surface energies of the sample and the probe liquid and the superscripts d and p refer to the dispersion and polar components of the surface energy, respectively. The polar energy was 32.3, 25.9, 40.1, 40.0 mJ/m^2, and the dispersive energy was 31.5, 34.8, 34.8, 34.8 mJ/m^2 for ITO film, Ag NPs, ITO BRs, and ITO BRs/Ag NPs,

respectively (Figure S8c). It can be found that the total surface energy was a little larger on ITO BRs (74.9 mJ/m^2) and ITO BRs/Ag NPs (74.8 mJ/m^2) than ITO film (63.8 mJ/m^2) and ITO Ag NPs (60.7 mJ/m^2). Also, the coating of Ag NPs slightly decreased the surface energy of ITO film and ITO BRs, but there is no significant difference.

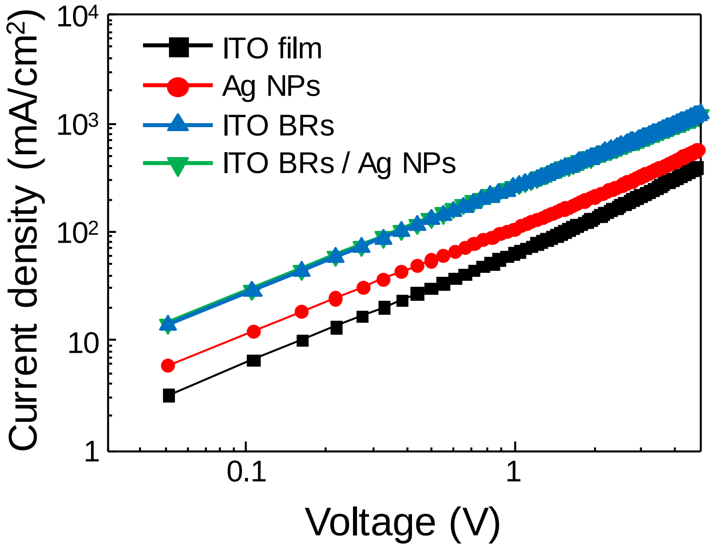

Figure S9. Space-charge limited current (SCLC) of hole-only device of electrodes/PEDOT:PSS/PTB7:PCBM/Au. Electrodes of ITO film, Ag NPs, ITO BRs and ITO BRs/Ag NPs were conducted for the evaluation.

In order to investigate whether the surface energy of electrodes affects the charge carrier mobility of PTB7:PCBM active layer, we measured the hole mobility of PTB7:PCBM active layer by measuring the space-charge limited current (SCLC) of the hole-only devices of electrodes/PEDOT:PSS/PTB7:PCBM/Au (Figure S9) in dark condition. Assuming ohmic contact and trap free transport, mobility from SCLC can be calculated by Mott–Gurney's equation: [S4]

$$J = \frac{9}{8}\varepsilon_r \varepsilon_0 \mu \frac{V^2}{L^3}$$

where J is the current density, ε_r is the dielectric constant of the organic semiconductor (generally 3), ε_0 is the permittivity of free space, μ is the mobility, V is the voltage, and L is the thickness of the active layer. The mobility of PTB7:PCBM calculated from the above equation is of the order of $\sim 10^{-3}$. All the devices showed nearly similar hole mobility regardless of the type of electrodes, indicating that the surface energy of electrodes does not change the charge carrier mobility of PTB7:PCBM active layer. Also, the trends in current

density (ITO BRs/Ag NPs ≈ ITO BRs > Ag NPs > ITO film) evidences that the increased surface roughness by Ag NPs and ITO BRs improved the electrical properties by reducing the resistance between the electrodes and PTB7:PCBM layers. From these results, we believe that the change in electronic properties of the PTB7: PCBM layers are not noticeable, but the electrical resistance between ITO BRs and the active layer is improved possibly by increased contact area.

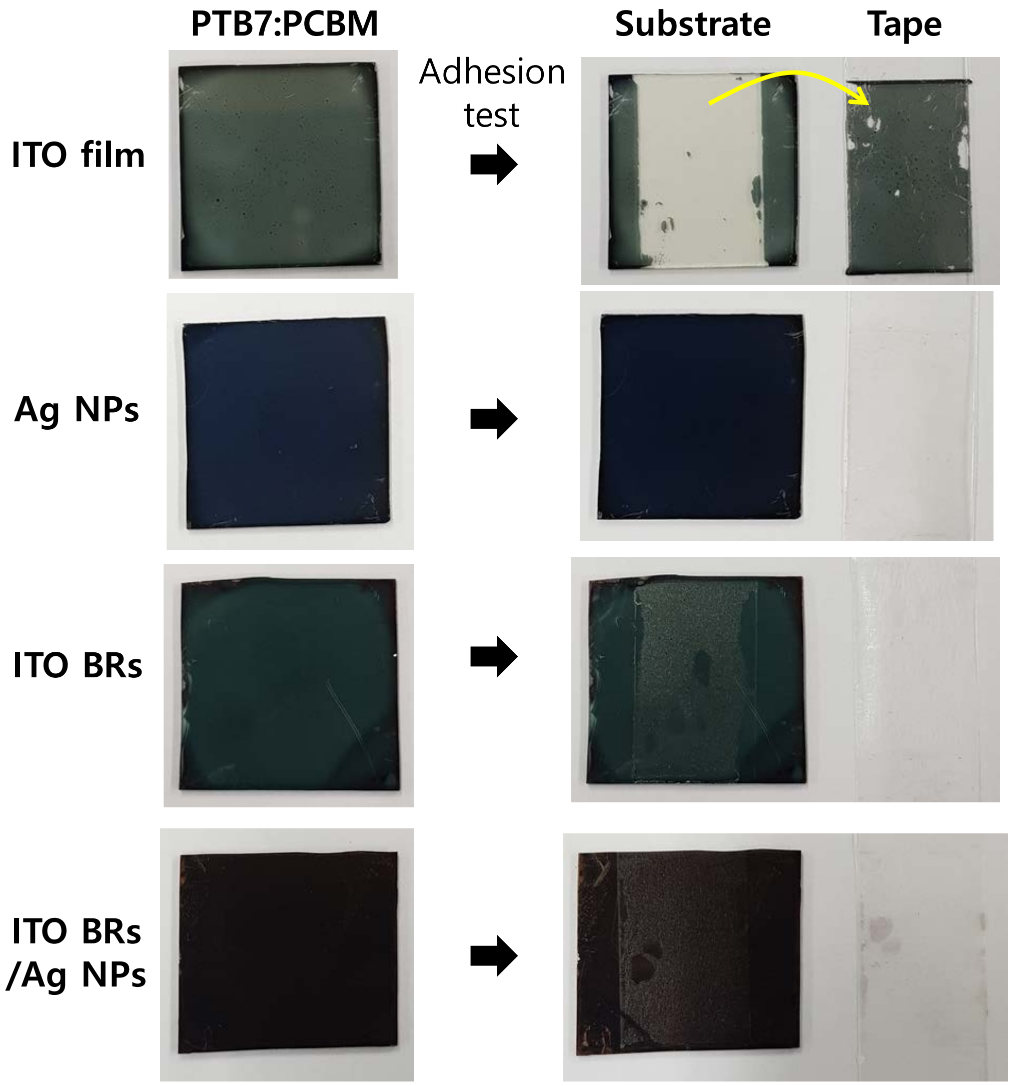

Figure S10. Photographic images of PTB7:PCBM polymer layers coated on the various transparent electrodes of ITO film, Ag NPs, ITO BRs and ITO BRs/Ag NPs (left). Adhesion of the polymer layers on the electrodes was evaluated by attaching and detaching an adhesive tape (right).

We further conducted experiments to demonstrate whether the surface energy of electrodes affects the adhesive strength of PTB7:PCBM active layer on the electrodes. PTB7: PCBM

layer coated on the electrodes of ITO film, Ag NPs, ITO BRs and ITO BRs/Ag NPs. Then, the adhesion strength of the PTB7:PCBM layers on the electrodes was evaluated by attaching and detaching an adhesive tape (Figure S10). It is noticeable that PTB7:PCBM layer was peeled off from the ITO film and transferred to the adhesive tape. But, PTB7:PCBM layers coated on Ag NPs, ITO BRs and ITO BRs/Ag NPs electrodes were not peeled off from the substrate. In addition, the adhesive glue was transferred from tape to ITO BRs and ITO BRs/Ag NPs. From these results, we found that the physical adhesion between the electrode and the polymer layer is more dependent on nanostructures in between the electrode and active layer rather than the surface energy. The nanostructures between the electrodes and the polymers are expected not only to improve adhesion but also to improve electrical properties by increasing the contact area between the electrode and the active layer.

Optical simulations of localized electric field enhancement near Ag NPs

λ	Structure	TM	TE
450 nm	ITO BRs	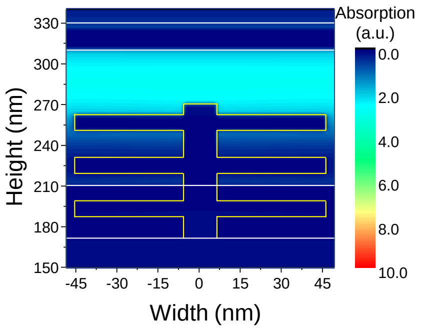	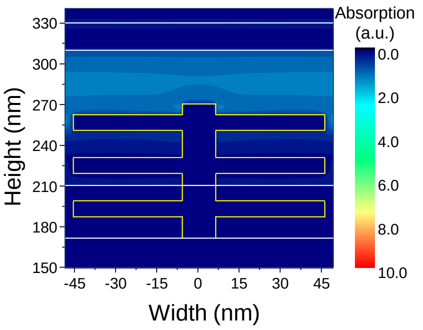
	ITO BRs /Ag NPs	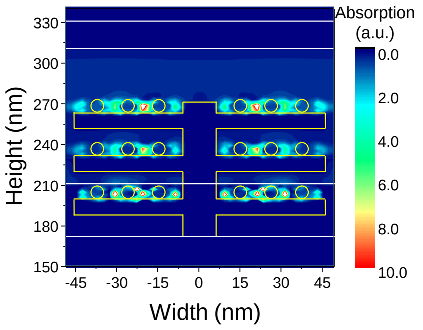	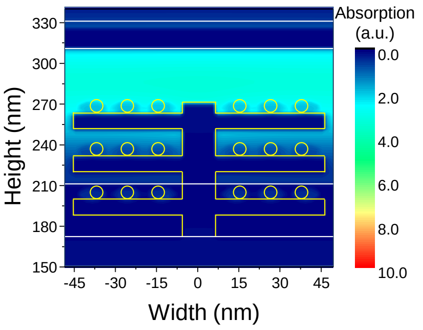
650 nm	ITO BRs	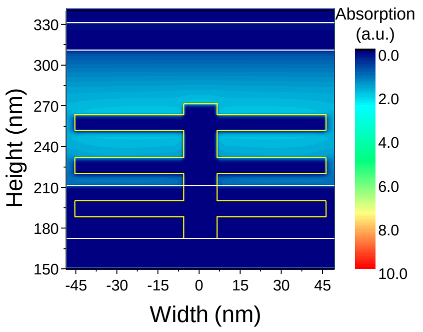	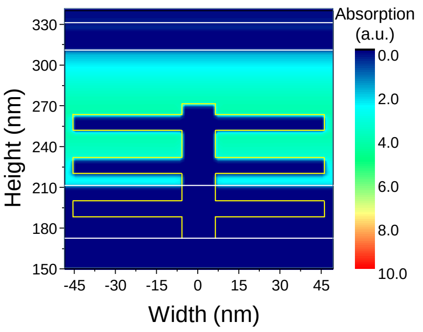
	ITO BRs /Ag NPs	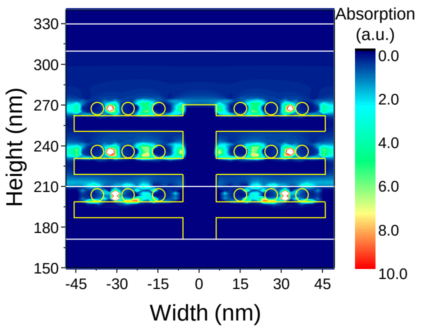	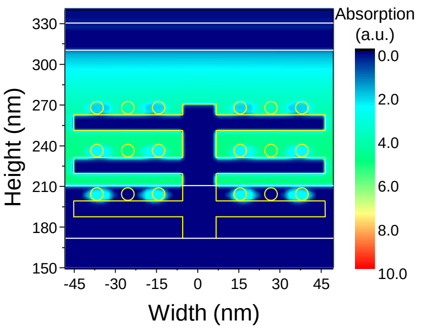

Figure S11. Distributions of light absorption in OSCs fabricated on ITO BRs and ITO BRs/Ag NPs. Two types of transverse magnetic (TM) and transverse electric (TE) waves were used as a light source for the simulation at the wavelength of 450 nm and 650 nm.

Distributions of light absorption in OSCs were simulated by using finite-domain time-difference (FDTD) optical simulator. Incident electromagnetic waves of transverse magnetic (TM) mode (right column) and transverse electric (TE) mode (left column) were illuminated on the surface normal direction at wavelength λ = 450 and 650 nm (Figure S11). Light absorption (U_{abs}) was converted from electric field according to the following equations:

$$U_{abs}(\vec{r},t) = 4\pi nk\left|E(\vec{r},t)\right|^2 / \lambda$$

Where n is refractive index, k is extinction coefficint. Because plasmonic Ag NPs only interact with TM waves, no electric field enhancement was observed with TE waves in all samples. Also, TM waves showed no local electric field enhancement in ITO BRs because there was no plasmonic NPs. On the other hand, since plasmonic electric-field near Ag NPs interacts with each other, a strong electric field was localized between the Ag NPs. The localized plasmonic field enhancement was not only strong at λ = 450 nm, but also strong at λ = 650 nm. Based on this result, ITO BRs/Ag NPs are found to be a good 3D plasmonic nanostructure for broadband light absorption in organic solar cells.

Energy band diagram of polymer solar cells

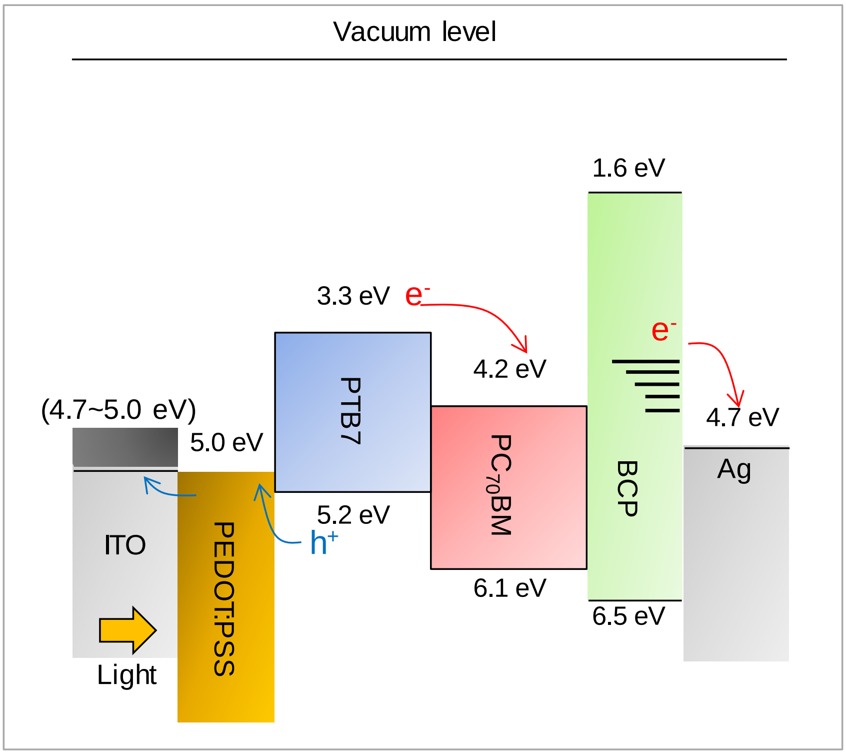

Figure S12. Energy band diagram of PTB7:PCBM bulk-heterojunction solar cells.

Energy diagram of each layer in PTB7:PCBM solar cell was illustrated (Figure S12). The highest occupied molecular orbital and lowest unoccupied molecular orbital levels of ITO, PEDOT:PSS, PTB7, $PC_{70}BM$, BCP and thin Ag electrode were obtained from the literatures.[S5,S6] The energy diagram confirms that the energy levels between ITO (4.7~5.0 eV) and PTB7 (5.2 eV) could be well-aligned by incorporation PEDOT:PSS hole transport layer (5.0 eV).

References

[S1] H. A. Atwater, A. Polman, *Nature Mater.* **2010**, *9*, 211.

[S2] T. T. Chau, W. J. Bruckard, P. T. L. Koh, A. V. Nguyen, *Adv. Colloid Interface Sci.* **2009**, *150*, 106.

[S3] D. K. Owens, R. C. Wendt, *Appl. Polymer Sci.* **1969**, *13*, 1741.

[S4] Z. B. Wang, M. G. Helander, M. T. Greiner, J. Qiu, Z. H. Lu, *J. Appl. Phys.* **2010**, *107*, 034506.

[S5] S. Woo, W. H. Kim, H. Kim, Y. Yi, H. –K. Lyu, Y. Kim, *Adv. Energy. Mater.* **2014**, *4*, 1301692.

[S6] G. H. Jung, K. Hong, W. J. Dong, S. Kim, J. –L. Lee, *Adv. Energy Mater.* **2011**, *1*, 1023.
